# Serological and genetic complement alterations in infection-induced and complement-mediated hemolytic uremic syndrome

**DOI:** 10.1007/s00467-016-3496-0

**Published:** 2016-10-07

**Authors:** Dineke Westra, Elena B. Volokhina, Renate G. van der Molen, Thea J. A. M. van der Velden, Annelies Jeronimus-Klaasen, Joop Goertz, Valentina Gracchi, Eiske M. Dorresteijn, Antonia H. M. Bouts, Mandy G. Keijzer-Veen, Joanna A. E. van Wijk, Jaap A. Bakker, Anja Roos, Lambert P. van den Heuvel, Nicole C. A. J. van de Kar

**Affiliations:** 1 Department of Pediatric Nephrology (804), Radboud University Medical Center, P.O. Box 9101, 6500 HB Nijmegen, The Netherlands; 2 Department of Laboratory Medicine, Radboud University Medical Center, Nijmegen, The Netherlands; 3 Department of Pediatric Nephrology, University Medical Center Groningen, Groningen, The Netherlands; 4 Department of Pediatric Nephrology, Erasmus MC - Sophia Children’s Hospital, Rotterdam, The Netherlands; 5 Department of Pediatric Nephrology, Academic Medical Center, Amsterdam, The Netherlands; 6 Department of Pediatric Nephrology, University Medical Centre Utrecht, Utrecht, The Netherlands; 7 Department of Pediatric Nephrology, VU University Medical Center, Amsterdam, The Netherlands; 8 Department of Clinical Chemistry and Laboratory Medicine, Leiden University Medical Centre, Leiden, The Netherlands; 9 Department of Medical Microbiology and Immunology, Sint Antonius Hospital, Nieuwegein, The Netherlands; 10 Department of Pediatrics, Department of Growth and Regeneration, University Hospital Leuven, Leuven, Belgium

**Keywords:** Atypical HUS, Complement, Hemolytic uremic syndrome, STEC, Genetic aberrations, Children

## Abstract

**Background:**

The role of complement in the atypical form of hemolytic uremic syndrome (aHUS) has been investigated extensively in recent years. As the HUS-associated bacteria Shiga-toxin-producing *Escherichia coli* (STEC) can evade the complement system, we hypothesized that complement dysregulation is also important in infection-induced HUS.

**Methods:**

Serological profiles (C3, FH, FI, AP activity, C3d, C3bBbP, C3b/c, TCC, αFH) and genetic profiles (*CFH*, *CFI*, *CD46*, *CFB*, *C3*) of the alternative complement pathway were prospectively determined in the acute and convalescent phase of disease in children newly diagnosed with STEC-HUS or aHUS. Serological profiles were compared with those of 90 age-matched controls.

**Results:**

Thirty-seven patients were studied (26 STEC-HUS, 11 aHUS). In 39 % of them, including 28 % of STEC-HUS patients, we identified a genetic and/or acquired complement abnormality. In all patient groups, the levels of investigated alternative pathway (AP) activation markers were elevated in the acute phase and normalized in remission. The levels were significantly higher in aHUS than in STEC-HUS patients.

**Conclusions:**

In both infection-induced HUS and aHUS patients, complement is activated in the acute phase of the disease but not during remission. The C3d/C3 ratio displayed the best discrepancy between acute and convalescent phase and between STEC-HUS and aHUS and might therefore be used as a biomarker in disease diagnosis and monitoring. The presence of aberrations in the alternative complement pathway in STEC-HUS patients was remarkable, as well.

**Electronic supplementary material:**

The online version of this article (doi:10.1007/s00467-016-3496-0) contains supplementary material, which is available to authorized users.

## Introduction

The hemolytic uremic syndrome (HUS) is a rare and severe disease characterized by the triad of hemolytic anemia, thrombocytopenia, and acute renal failure. HUS is characterized histologically by thrombotic microangiopathy (TMA): vascular abnormalities with glomerular endothelial damage, swelling of the endothelium, endothelial detachment of the basement membrane, intima fibrosis, and thrombosis [1]. In recent years, knowledge of the pathogenesis of HUS has extended tremendously [2]. In >90 % of cases, the disease is triggered by an infection with Shiga-like toxin-producing *Escherichia coli* (STEC-HUS). Non-STEC-HUS patients have a poor prognosis, with high mortality and morbidity rates in the acute phase and progression to end-stage renal disease (ESRD) in 50 % of cases. Several causes of this non-STEC-HUS have been identified, including disorders of complement regulation (aHUS) and various nonenteric infections, such as *Streptococcus pneumoniae* (SP-HUS) [3].

**Fig. 1:**
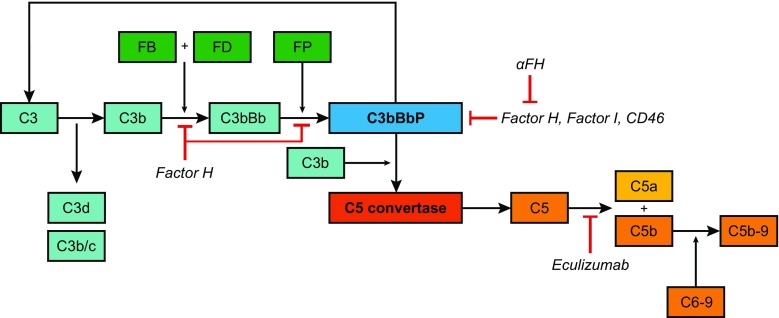
Alternative and terminal pathway of the complement system. The central complement component C3 is spontaneously activated at a very low rate to C3b, thereby generating the C3 breakdown products C3d and C3b/c. C3b is able to attach to the surfaces of pathogens and host cells. There it binds complement factor B (FB), which is proteolytically activated by factor D (FD). The resulting C3bBb complex is stabilized by properdin (FP), and this C3 convertase can cleave and activate more C3 molecules. This activation leads to amplification of the complement cascade via the C5 convertase, to the formation of the membrane attack complex (C5b-9 or TCC), and, eventually, to cell lysis. The regulators of the complement system, important in the protection of host cells against complement activation, and the complement-inhibiting therapeutic eculizumab, are depicted at the level of action

Dysregulation of the alternative pathway (AP) of the complement system plays an important role in the pathogenesis of aHUS. Figure 1 presents a schematic overview of the alternative and terminal complement pathways. In recent years, DNA mutation analysis of genes encoding complement proteins in patients with aHUS have clearly demonstrated that in 50–60 % of patients, mutations are found in *CFH*, *CFI*, *CD46*, *CFB*, *C3*, and *THBD* [4–11]. A subgroup of patients with aHUS has been described as having antibodies against the C-terminus of FH (αFH) in combination with a polymorphic homozygous deletion of the genes encoding complement-factor-H-related proteins 1 and 3 (*CFHR1*/*3*) [12]. Aside from the aforementioned genes, mutations have been identified in two genes encoding coagulation proteins (*DGKE* and *PLG*) [13, 14] and in cobalamin C (*MMACHC*) in combination with complement aberrations [15].

Recent in vitro studies have shown that the microbial pathogens associated with HUS—or their toxins—can activate the complement system and bind complement proteins. In this way, the organism can protect itself against complement activation [16–18]. Based on the similarity of the clinical manifestations of complement-mediated and infection-induced HUS and the knowledge that bacteria can use complement regulators to survive in the host, we hypothesized that also in patients with STEC-HUS, a dysregulation of the complement system has an important place in the pathogenesis.

Anecdotal reports have shown low C3 levels and increased complement activation products in children with STEC-HUS, but usually only a few activation markers were measured and in a small research population [19–23]. To answer the question of whether alterations in the alternative complement pathway are common in patients with all forms of HUS, we prospectively determined genetic and serological complement profiles in serum and plasma of children with STEC-HUS or aHUS in the acute and convalescent phase of the disease. Currently, in most studies, control ranges for adults are used to interpret results of serological complement tests in children, which is an important shortcoming in the correct interpretation of the results. Therefore, following a strict protocol, we screened complement profiles in age-matched, healthy Dutch infants and children to obtain reliable reference intervals by which to interpret our studies in HUS children.

## Methods

### Patients and controls

Newly diagnosed children (0–18 years) with STEC-HUS or aHUS referred to the pediatric nephrology ward of all academic hospitals in The Netherlands between August 2010 and September 2013 were eligible for enrolment. In The Netherlands, the policy is to refer every suspected HUS patients to the academic setting. We expect that only a very low proportion (approximately 5–10 %) of STEC-HUS patients were not referred and would not have been assessed in this study. HUS was diagnosed based on the triad of microangiopathic hemolytic anemia, thrombocytopenia, and acute renal failure; patients were further classified as STEC-HUS, SP-HUS, or aHUS, as previously described [24]. STEC-HUS cases were confirmed by stool samples [culture and/or stx1 and stx2 detection by polymerase chain reaction/enzyme immunoassay (PCR/EIA)].

Ethylenediaminetetraacetic acid (EDTA) plasma and/or serum samples were collected before the initiation of therapy [acute-phase sample; usually 3–7 days after onset of gastrointestinal (GI) complaints] and 14–28 days later (convalescent phase sample), preferably when all signs of TMA had disappeared. To obtain a reliable control population, 90 pediatric individuals of different ages were enrolled in the study to assess the correlation between complement system and age. Exclusion criteria were fever (>38.5 °C), signs or symptoms of infection (bacterial or viral), chronic illness, immune-suppressive medication, acquired or congenital immune deficiencies, age <2 days of life, intensive ventilation, and surgical interventions in last 3 days.

### Sample collection

For serological complement profiling, EDTA blood samples were placed on ice immediately after collection and were processed within 1 h (10 min, 2500 g, 4 °C); whole-blood samples were allowed to coagulate for 45–60 min before processing (10 min, 2500 g, 4 °C). Serum and EDTA plasma samples were stored at −80 °C in aliquots.

For DNA analysis, genomic DNA was isolated from peripheral blood leukocytes according to established protocols. For one STEC-HUS patient, no material was available for genomic DNA isolation.

### Genetic analysis

Genomic DNA was amplified for *CFH* [National Centre for Biotechnology Information (NCBI) RefSeq NM_000186.3], *CFI* (NM_000204.3), *CD46* (NM_002389.4), *C3* (NM_000064.2), and *CFB* (NM_001710.5) by means of PCR, as previously described [8]. Primer data are available upon request. Nonsynonymous aberrations were checked in the literature, evolutionairy conservation (USCS Genome Browser; http://genome.ucsc.edu), in silico prediction programs [Sorting Intolerant From Tolerant (SIFT); http://sift.jcvi.org/; a <0.05 was considered not tolerated], and PolyPhen-2 (Polymorphism Phenotyping v2; http://genetics.bwh.harvard.edu/pph2/). We also searched in the Exome Variant Server (http://evs.gs.washington.edu/EVS/), the Exome Aggregation Consortium (ExAC, http://exac.broadinstitute.org/), and in an in-house database, which all contain results of whole-exome sequencing of >5000 individuals of European background (non-Finnish for the ExAC database).

### Presence of autoantibodies against factor H and anti-O157 LPS antibodies

Acute-phase serum samples were tested for the presence of αFH and anti-O157 lipopolysaccharide (LPS) by means of enzyme-linked immunosorbent assay (ELISA), as described previously [25, 26]. LPS from *E. coli* O157:H7 for coating was obtained via List Biological Laboratories (Campbell, CA, USA).

### Serological complement profiling of the alternative complement pathway

The C3 concentration was determined by nephelometry (BN™ II System, Siemens Healthcare Diagnostics, Erlangen, Germany) using reagents from Beckman Coulter Inc (Brae, CA, USA); levels were standardized against the ERM‐DA470k/IFCC serum [27]. Levels of FH and FI were determined by radial immunodiffusion. For FH, a rabbit antiserum raised against purified human factor H was used. Calibration was based on serial dilutions of normal human serum with a known concentration of FH (in mg/L). For FI, a goat antiserum raised against purified human factor I was used. Calibration was based on serial dilutions of normal human serum and expressed as percentage of the value in this standard human serum (% NHS). To analyze AP activity, a commercially available ELISA kit for total functional assessment of the AP was used according to the manufacturer’s protocol (Euro Diagnostica, Malmö, Sweden).

Levels of the C3 degradation products C3d and C3b/c and the AP convertase C3bBbP were quantified in EDTA plasma using ELISA, as previously described in detail [28, 29]. As the initial C3 concentration may influence the C3d level, the C3d/C3 ratio was calculated as an extra marker of AP activation independent of the concentrations of individual molecules. For this matter, C3d levels were multiplied by 100 and divided by C3 levels. The fluid-phase terminal complement complex (TCC) was measured using a commercially available ELISA kit (Hycult Biotech, Uden, The Netherlands), according to the manufacturer’s protocol.

### Statistical analysis

A linear regression analysis was performed for the control group to investigate the possible correlation between age and complement profiles. For each investigation, a D’Agostino-Pearson normality test was executed to assess whether the controls were sampled from a Gaussian distribution. When controls were normally distributed, a one-way analysis of variance (ANOVA) with a Dunnett’s post test was executed to analyze differences between controls and independent patient groups; in case controls did not pass the normality test, a one-way ANOVA with a Dunns’ post-test was performed. A Mann–Whitney test was used to investigate the difference in age between STEC-HUS and aHUS patients and to compare between the acute and convalescent phase in these independent patient groups. To compare C3d/C3 ratios between patient groups in the acute phase of disease, a Pearson’s chi-square test was performed. All statistical analyses were performed using GraphPad PRISM software (version 5.03 for Windows, GraphPad Software), except for the Pearson’s chi-square test, which was executed in SPSS (version 20, IBM).

## Results

### Clinical characteristics of included patients

**Fig. 2:**
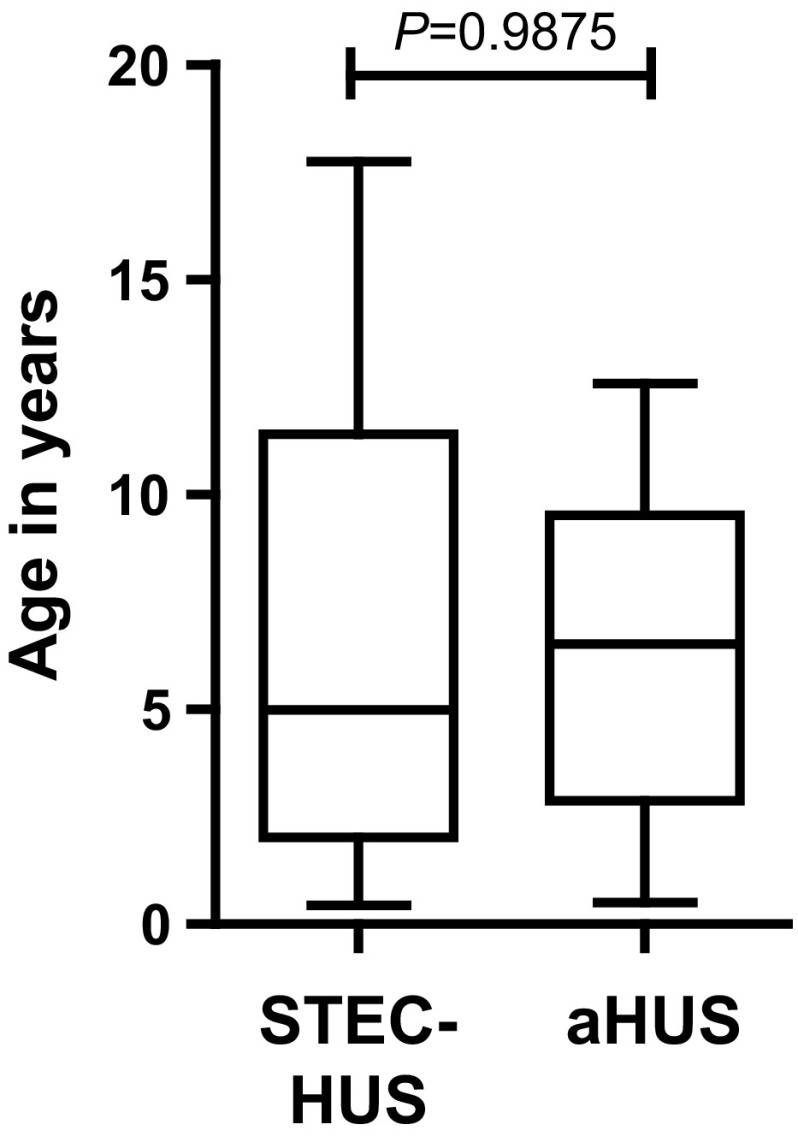
Age distribution of Shiga-toxin-procuding *Escherichia coli*-induced hemolytic uremic syndrome (STEC-HUS) and atypical HUS (aHUS) patients at time of diagnosis. No statistically significant difference is seen between STEC-HUS and aHUS patients. *Thick horizontal line in box* represents median; *top and bottom of box*: first and third quartiles (25th–75th percentile);* whiskers*: min to max

Thirty-seven patients, all with a Caucasian background, were enrolled in this study between 2010 and 2013: 26 STEC-HUS patients and 11 aHUS patients. Epidemiological features, genetic results, and clinical characteristics (presenting symptoms, treatment, and outcome) for 35 patients (94.6 %) are listed in Table 1. The clinical courses of two patients (P1 and P6) have been extensively described [30, 31]. Mean age of all patients at presentation was 6.6 years (6.6 years for STEC-HUS; 6.5 years for aHUS). No significant difference was seen in age at presentation between STEC-HUS and aHUS patients (*P* = 0.9875; Fig. 2). Even though in the literature it is stated that STEC-HUS is usually diagnosed in children between 2 and 5 years of age, in our study, half of the STEC-HUS patients (13/26; 50.0 %) were >5 years of age (range 0.4–17.8 years).

**Table 1:** Epidemiological and clinical features of studied patients

Feature	All patients	STEC-HUS	aHUS
Number	37	26 (68.4 %)	11 (28.9 %)
Gender	20 M : 17 F	13 M : 13 F	7 M : 4 F
Age (years) mean ± SD (range)	6.6 ± 4.8 (0.4–17.8)	6.6 ± 5.3 (0.4–17.8)	6.5 ± 3.8 (0.5–12.6)
Follow-up (years) mean ± SD (range)	2.9 ± 0.9 (1.3–4.4)	2.7 ± 0.9 (1.3–4.4)	3.1 ± 1.0 (1.9–4.3)
Serotype	Not applicable	O157: 16 (61.5 %)	Not applicable
		O26: 4 (15.4 %)	
		O5: 1 (3.8 %)	
		O104: 1 (3.8 %)	
		Not serotyped, but α-O157 negative: 4 (15.4 %)	
Presentation			
Diarrhea	14 (34)	12 (23)	2 (11)
Bloody diarrhea	9 (34)	9 (23)	0 (11)
Gastrointestinal symptoms without diarrhea	8 (34)	2 (23)	6 (11)
Upper respiratory tract infection	4 (34)	1 (23)	3 (11)
Oligo/anuria	14 (34)	8 (23)	6 (11)
Headache	1 (34)	0 (23)	1 (11)
Extrarenal complications			
None	27 (34)	16 (23)	11 (11)
Yes	7 (34)	7 (23)	0 (11)
Neurological	7 (34)	7 (23)	0 (11)
Intestinal (sigmoid stenosis, peritonitis)	1 (34)	1 (23)	0 (11)
Chronic venous insufficiency thrombosis	1 (34)	1 (23)	0 (11)
Treatment			
Spontaneous remission	9 (34)	8 (23)	1 (11)
Dialysis	13 (34)	13 (23)	0 (11)
Plasma therapy	3 (34)	0 (23)	3 (11)
Dialysis and plasma therapy	9 (34)	2 (23)	7 (11)
Eculizumab	5 (34)	2 (23)	3 (11)
Outcome			
Normal renal function	26 (33)	18 (23)	8 (10)
Hypertension^1^ and proteinuria^2^	8 (33)	3 (23)	5 (10)
Hypertension^1^	2 (33)	1 (23)	1 (10)
Proteinuria^2^	4 (33)	4 (23)	0 (10)
Maintenance treatment with eculizumab	2 (33)	0 (23)	2 (10)
Relapses after this episode	3 (33)	0 (23)	3 (10)
Genetic or acquired complement aberrations (see Table 2)			
Total	14/36 (38.9 %)	7/25 (28.0 %)	7/11 (63.3 %)
*CFH*	2/36 (5.6 %)	2/25 (8.0 %)	–
*CD46*	2/36 (5.6 %)	–	2/11 (18.2 %)
*C3*	3/36 (8.3 %)	2/25 (8.0 %)	1/11 (9.1 %)
*C3* and αFH	1/36 (2.8 %)	–	1/11 (9.1 %)
αFH	6/36 (16.7 %)	3/25 (12.0 %)	3/11 (27.3 %)

For clinical features, the numbers of patients with data available are reported in parentheses

*αFH* autoantibodies against factor H, *aHUS* atypical HUS, *C3* complement component 3, *CD46* membrane cofactor protein, *CFH* complement factor H, *HUS* hemolytic uremic syndrome, *SD* standard deviation, *STEC*-*HUS* Shiga-toxin producing *Escherichia coli*-induced HUS

^1^Hypertension: a systolic and/ or diastolic pressure ≥ 2.0 standard deviation scores compared with normal values for age, gender, and height

^2^Proteinuria: > 2 years old > 0.2 mg/mg (>22.6 mg/ mmol or 0.226 g/10 mmol); < 2 years old > 0.5 mg/mg (>56.6 mg/mmol or 0.566 g/10 mmol)

Moe than 60 % of the STEC-HUS cases (16/26) were preceded by a proven infection with STEC O157. In four patients (15.4 %), culture and/or PCR was positive for STEC-infection, but no serotype could be identified; these patients were all negative for anti-O157 LPS antibodies. The remaining cases were infected with STEC O26, O5, or O104. All STEC-HUS patients presented with (bloody) diarrhea and/or gastrointestinal complaints; one patient also had an upper respiratory tract infection. Neurological complications were seen in seven STEC-HUS patients (7/23; 30.4 %). Two of these patients had other extrarenal complications as well: one had chronic venous insufficiency during the HUS episode, and the other was diagnosed with sigmoid stenosis due to HUS 3 months after discharge, for which a sigmoid resection and end-to-end anastomosis was needed [30]. Dialysis was needed in 65.2 % (15/23) of the STEC-HUS patients (average duration 16 days); the other patients recovered without the need for renal replacement therapy. Two STEC-HUS patient were treated with plasmapheresis subsequent to dialysis. In one of them, plasmapheresis was indicated because of anuria, symptoms of ileus, and severity of neurological condition; plasma therapy was stopped when an STEC O26 infection was confirmed. Two STEC-HUS patients were treated with eculizumab: one STEC O104 patient was treated for 8 weeks in the ad hoc, off-label trial during the German outbreak; the other had such an unusual presentation (12 years old, upper respiratory tract infection, no bloody diarrhea) that aHUS was suspected and one dose of eculizumab was given before anti-O157 LPS antibodies were identified. The majority of STEC-HUS patients had a good outcome with normal renal function, although hypertension and/or proteinuria were still present after >1 year following the acute phase in eight patients (8/23; 34.8 %).

Interestingly, 70 % of aHUS patients presented with GI complaints: 18 % with diarrhea and 54 % with other GI symptoms, such as stomach aches and vomiting. No bloody diarrhea was seen in aHUS patients. No extrarenal complications were seen in these patients, and the majority were treated with both dialysis (average duration 9 days) and plasma therapy (average number of sessions 14) to control renal failure. Sequelae were still present in 70 % of aHUS patients, in most cases both hypertension and proteinuria. For three aHUS patients, the HUS episode at the time of the study was a recurrence. In one of these patients (P20), the first episode was thought to be induced by an STEC infection based on presentation with bloody diarrhea. One aHUS patient (P24) was enrolled in the pediatric eculizumab trial [32], and a third, with a recurrence, was treated with eculizumab outside the pediatric trial. Eculizumab treatment had to be discontinued in both patients when the trial ended, after which a relapse of disease occurred. These patients are now on maintenance treatment with eculizumab.

### Genetic and/or acquired complement aberrations in HUS patients

All but one patient in the study were screened for mutations in the complement genes *CFH*, *CFI*, *CD46*, *CFB*, and *C3*, all of which are associated with aHUS, and for the presence of αFH. In 38.9 % (14/36) of the patients, including 7/25 STEC-HUS patients, we identified a genetic and/or acquired complement abnormality. Characteristics of the identified mutations are depicted in Table 2.

**Table 2:** Characteristics of genetic and acquired complement aberrations identified in the enrolled STEC-HUS, SP-HUS, and aHUS patients

	Disease	Complement aberration	SIFT	PolyPhen	EVS	IHD	ExAC	Literature
P9	STEC-HUS	αFH	–	–	N.A.	N.A.	–	HUS: Jozsi et al.[12]
P10	STEC-HUS	αFH	–	–	N.A.	N.A.	–	HUS: Jozsi et al.[12]
P14	STEC-HUS	*C3*: p.Arg1219His	Tolerated	Benign	1/6503 (0.02 %)	0/5036 (0.00 %)	1/66732 (0.01 %)	–
P21	STEC-HUS	*CFH*: p.Thr956Met	Tolerated	Probably damaging	15/6503 (0.23 %)	1/5036 (0.02 %)	87/66734 (0.13 %)	HUS: Perkins, Goodship.[45]
P25	STEC-HUS	*C3*: p.Lys155Gln	Tolerated	Benign	38/6503 (0.58 %)	34/5036 (0.68 %)	395/66700 (0.59 %)	AMD: Seddon et al.[44]
P29	STEC-HUS	*CFH*: p.Ser58Ala	Tolerated	Benign	2/6503 (0.03 %)	0/5036 (0.00 %)	11/66526 (0.02 %)	–
P32	STEC-HUS	αFH	–	–	N.A.	N.A.	–	HUS: Jozsi et al.[12]
P3	aHUS	αFH	–	–	N.A.	N.A.	–	HUS: Jozsi et al.[12]
P8	aHUS	*C3*: p.Arg161Trp; αFH	Deleterious	Probably damaging	0/6503 (0.00 %)	6/5036 (0.12 %)	Not present	HUS: Volokhina et al.[9]
P18	aHUS	αFH	–	–	N.A.	N.A.	–	HUS: Jozsi et al.[12]
P20	aHUS	*CD46*: p.Asp271_Ser272del	–	–	0/6503 (0.00 %)	1/5036 (0.02 %)	Not present	HUS: Richards et al.[10]
P31	aHUS	αFH	–	–	N.A.	N.A.	–	HUS: Jozsi et al.[12]
P33	aHUS	*CD46*: p.Cys35Tyr	Deleterious	Probably damaging	0/6503 (0.0 %)	2/5036 (0.04 %)	0/66592 (0.00 %)	HUS: Caprioli et al.[5]
P35	aHUS	*C3*: p.Arg161Trp	Deleterious	Probably damaging	0/6503 (0.00 %)	6/5036 (0.12 %)	Not present	HUS: Volokhina et al.[9]

*αFH* autoantibodies against factor H, *aHUS* atypical hemolytic uremic syndrome,* SP-HUS* *Streptococcus pneumoniae* HUS, *AMD* age-related macula degeneration, *C3* complement component 3, *CD46* membrane cofactor protein, *CFH* complement factor H, *EVS* Exome variant server (http://evs.gs.washington.edu/EVS/), *ExAC* Exome Aggregation Consortium (http://exac.broadinstitute.org/), *HUS* hemolytic uremic syndrome, *IHD* in-house database, *N.A*. not applicable, *N/D* not determined, *SIFT* Sorting Intolerant From Tolerant (http://sift.jcvi.org/), *PolyPhen* Polymorphism Phenotyping v2 (http://genetics.bwh.harvard.edu/pph2/), *STEC-HUS* Shiga-toxin-procuding *Escherichia coli*-induced HUS

### Correlation between serological complement profiles and age in healthy children

**Fig. 3:**
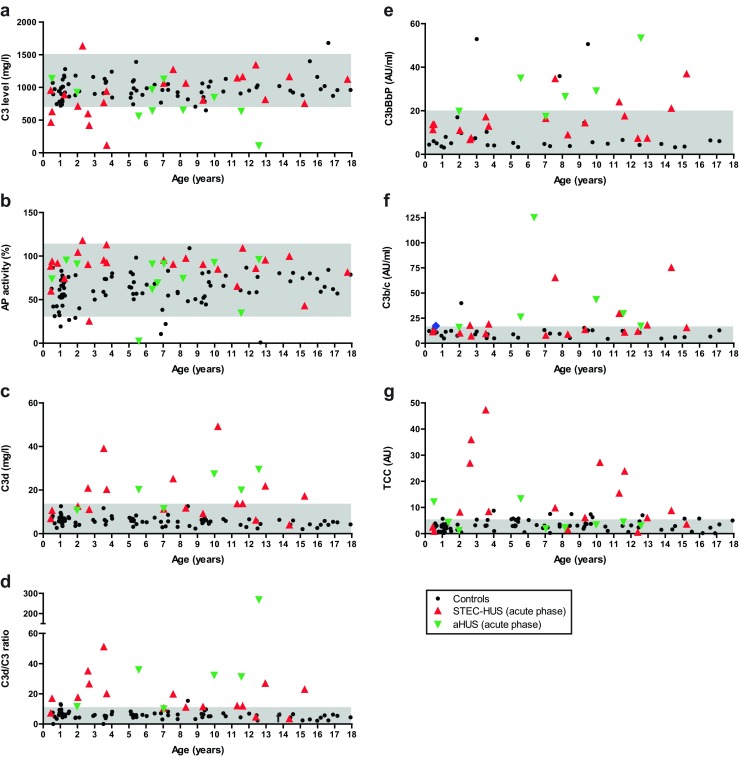
Serological complement profiles in individual controls and patients with Shiga-toxin-procuding *Escherichia coli*-induced hemlytic uremic syndrome (STEC-HUS) and atypical HUS (aHUS) in the acute phase of disease. Serum or plasma samples of 90 controls (aged 4 months to 18 years) and of HUS patients in the acute phase were analyzed for levels of alternative pathway (AP) proteins C3 (**a**), AP activity (**b**), and complement activation markers C3d, the C3d/C3 ratio, C3bBbP, C3b/c, and TCC (**c**–**g**). Each* symbol* indicates an individual control or patient; controls are symbolized with *black dots*, STEC-HUS patients with *red triangles*, and aHUS patients with *green triangles*. Control ranges of adult individuals for C3 (700–1500 mg/l), AP activity (30–113 %), C3d (<3.3 %), and TCC (<5 AU) levels are shown in *grey*. No adult reference ranges are available for the C3d/C3 ratio, C3bBbP, and C3b/c, but the reference interval as defined in our study (mean + two standard deviations of pediatric control values), is depicted in *grey*.*TCC* terminal complement component

We included 90 control patients (179 days to 18 years old) to define the correlation between complement levels/activity and age. The individual profiles for controls and those of patients in the acute phase of disease are shown in Fig. 3. No correlation was seen in healthy children between age and serological complement profiles except for C3d levels, which slightly decreased with increasing age (*P* = 0.0017; R^2^ = 0.1086). All levels were within adult reference ranges, although FI levels for pediatric controls are around the lower limit of the adult reference interval. For comparison of complement profiles between healthy children and patients, results of pediatric controls were grouped.

### Serological complement profiles in STEC-HUS, aHUS, and SP-HUS patients

**Fig. 4:**
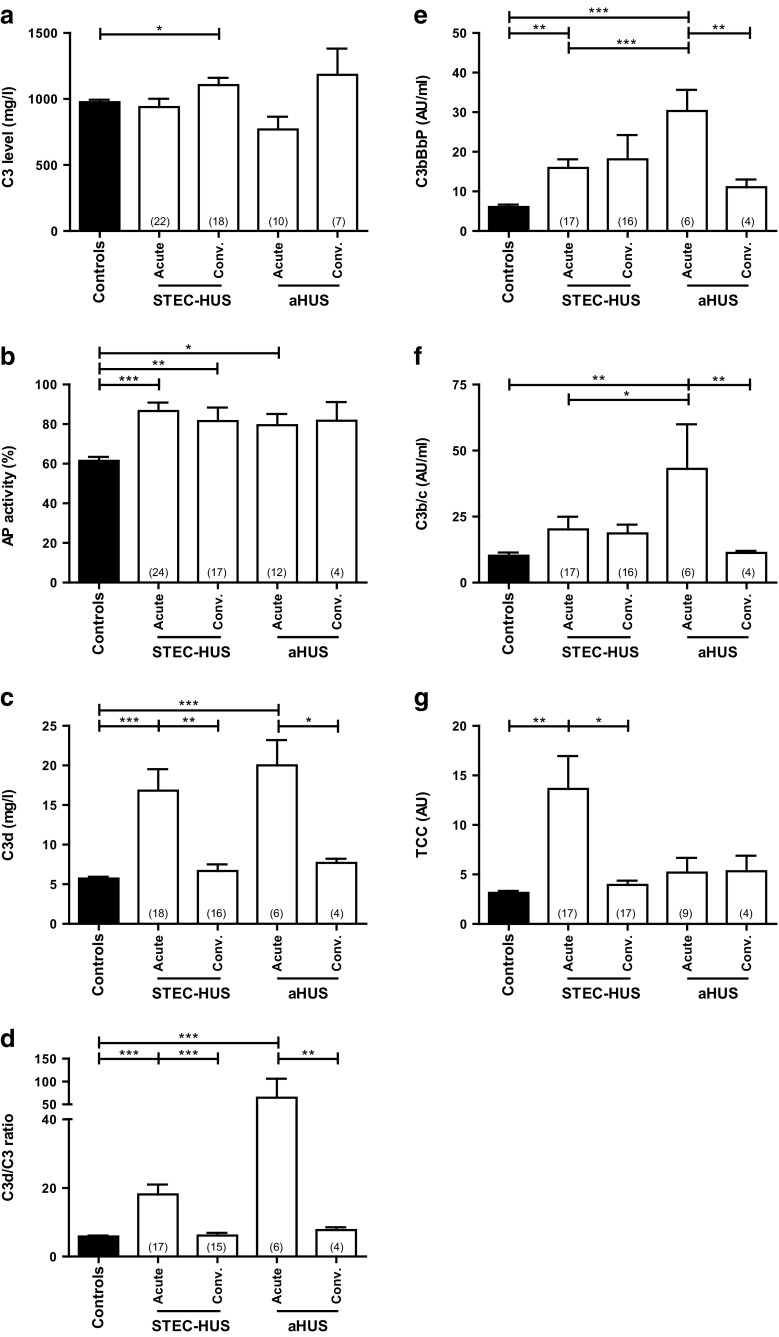
Serological levels of complement C3, alternative pathway activation, and levels of complement activation products in patients with Shiga-toxin producing *Escherichia coli*-induced hemolytic uremic syndrome (STEC-HUS) and atypical HUS (aHUS) in the acute and convalescent phases of disease. Serum or plasma samples taken in both phases were analyzed for the complement proteins C3 (**a**), for alternative pathway activation (**b**), and for the levels of C3d, C3d/C3 ratio, C3bBbP, C3b/c, and TCC (**c**–**g**). The number of screened patients is indicated within parentheses. * *P* < 0.05; **P < 0.01; ***P < 0.001. *Bars* mean; *error bars* standard error of mean. *AP* alternative pathway, *conv* convalescent, *TCC* terminal complement component

Levels of individual complement proteins C3, FH, and FI; AP activity, and the activation products C3d, C3bBbP, C3b/c, and TCC were measured in serum and EDTA plasma of HUS patients on admission and 14–28 days later. Results were compared with those of healthy pediatric controls (Fig. 3 and Electronic Supplementary Resource 1). In the acute phase, patients had slightly decreased complement C3 levels compared with pediatric age-matched controls, although the alterations were not significantly different between groups (Fig. 4a). These levels increased in remission in both STEC-HUS and aHUS patients. Levels of FH in STEC-HUS patients were significantly elevated compared with controls (*P* < 0.001 for the acute phase and *P* < 0.01 for the convalescent phase; data not shown); for FI levels, this was the case for all patient groups (*P* < 0.001; data not shown).

The mean AP activity in both phases was higher in patients than in controls, but this was not significantly different in all patient groups (Fig. 4d). Individual AP activity, however, was comparable with respective age-matched controls (Fig. 3d). In the acute phase, the levels of AP activation markers C3d (*P* < 0.001 for STEC-HUS and aHUS), C3bBbP (STEC-HUS: *P* < 0.01; aHUS: *P* < 0.001), and C3b/c (STEC-HUS: n.s.; aHUS: *P* < 0.01) were increased as well, as depicted in Fig. 4e, g, and h. The terminal AP marker TCC was only significantly increased in STEC-HUS patients (*P* < 0.01; Fig. 4i); TCC levels in aHUS and SP-HUS patients, however, were above the mean of pediatric controls as well, although not significantly. In all patient groups, levels of complement activation markers normalized to about control levels in the convalescent phase.

As C3d levels were age dependent, we calculated the C3d/C3 ratio as an extra marker of AP activation independent of initial C3 levels (Figs. 3f and 4f). This ratio showed no age-dependent effect. In the acute phase, the mean ratio in patients was more than three times higher than in controls (20.00 vs. 5.86; *P* = 0.0003). No significant difference was found between STEC-HUS and aHUS patients, but at a more detailed inspection of individual ratios, two thirds of aHUS patients had a C3d/C3 ratio >2.75, while 88 % of STEC-HUS patients had a ratio C<2.75 (*P* = 0.021). The other AP activation markers displayed a significant difference between STEC-HUS and aHUS patients in the acute phase of disease as well (C3bBbP: *P* = 0.011; C3b/c: *P* = 0.027).

The presence of a genetic aberration or αFH could not be linked to an altered serological complement profile: not all patients with a complement aberration displayed complement activation for the analyzed biomarkers in the acute and/or convalescent phase (Electronic Supplementary Resource 1). However, the power of this analysis was hampered by missing samples for analysis.

## Discussion

To investigate the role of the alternative complement pathway in HUS in general (infection-induced and aHUS), samples of 37 children with HUS of any etiology were collected in both the acute and convalescent phases of the disease. Levels of individual complement proteins and complement activation markers were measured. It was demonstrated that in the acute phase of the disease, both infection-induced and aHUS patients trended toward decreased average C3 levels and increased average AP activity, even though this difference was not statistically significant in all groups. Complement activation products C3bBbP and C3d and the C3d/C3 ratio were all significantly increased in STEC-HUS and aHUS patients. In aHUS patients, C3b/c was increased as well. This complement activation normalized to control levels in remission, except for C3bBbP in STEC-HUS patients. Our results corroborate previous reports of complement activation in children with STEC-HUS [19–23].

Thurman et al. [21] mention that measurement of TCC might be useful in monitoring the course of the STEC-HUS, and recent studies have shown that the activation products C3bBbP, C3b/c, and TCC can be used as biomarkers for disease activity in aHUS patients [33]. In our study, similar results were obtained in aHUS patients for C3bBbP and C3b/c, which all normalized in the convalescent phase, but not for TCC, levels of which levels did not change compared with the acute phase. Volokhina et al. used remission samples of patients who had their last aHUS episode more than 1 year previously, while our samples were collected within 1 month after the disappearance of clinical HUS symptoms. It seems that in aHUS, the terminal pathway remains activated for a longer period than the AP. No significant difference was seen between acute and convalescent C3bBbP and C3b/c levels in STEC-HUS patients.

In both patient groups, the C3d/C3 ratio gives the clearest difference between the acute and convalescent phases of disease in all patient groups (*P* < 0.01 STEC-HUS, *P* < 0.001 aHUS) and the C3d/C3 ratio may therefore be the most promising biomarker with which to monitor disease activity. The finding of increased complement activation products in the circulation during the acute phase of aHUS indicates that in this patient group, the AP might not only be dysregulated at the level of the glomerular endothelium, as was suggested in a recent study [34]. Noris et al. only measured activation products of the terminal pathway (TCC and C5a), but Volokhina et al. [33] and our study also investigated activation products of the AP. Our results with altered levels of C3bBbP, C3b/c, and the C3d/C3 ratio, but not TCC, show that increased complement turnover occurs, at least at the C3 level.

Based on clinical presentation, it sometimes can be difficult to distinguish between STEC-HUS and aHUS patients: no age differences were seen, many STEC-HUS cases were >5 years, and the majority of aHUS patients presented with gastrointestinal problems and/or diarrhea, which has been described before [35, 36]. Furthermore, not all STEC-HUS patients had (bloody) diarrhea, which again clearly shows that postdiarrheal onset does not exclude the possibility of aHUS or that the absence of diarrhea excludes STEC-HUS. The C3d/C3 ratio at admission was on average twice as high in aHUS patients than in infection-induced HUS patients; this was not a statistically significant difference, and was most probably due to the low number of patients. Based on visual analysis of the data plot, aHUS patients were more likely to have a C3d/C3 ratio >2.75 compared with STEC-HUS (66.7 vs. 11.8 %; *P* = 0.021). Levels of C3bBbP and C3b/c were also significantly higher in aHUS than in STEC-HUS patients. Based on our results, the investigated AP activation products may therefore be used as biomarkers to discriminate at admission between STEC-HUS and aHUS. As C3d is a more commonly performed measurement than C3bBbP and C3b/c, the C3d/C3 ratio is preferred, and this ratio may be used to monitor disease activity as well. The calculated ratio also compensates for the observed C3 consumption occurring in aHUS patients compared with controls. As the number of patients in the current study was very small, a prospective study is needed to determine sensitivity and specificity before these assays are implemented in routine diagnostics in case of HUS suspicion.

As the human complement system is one of first parts of innate immunity to be activated when micro-organisms invade the body, at this moment, we cannot exclude that the observed AP activation in STEC-HUS is only an infection-related phenomenon—for example, due to endotoxins or other proteins produced by the bacteria. In meningococcal infection, for instance, C3 activation and TCC are both increased, with a strong correlation between complement activation and levels of endotoxins produced by *Neisseria meningitidis* [37]. As the classic and lectin pathway could be activated as well due to the infection, we studied the classic pathway in both the acute and convalescent phase (data not shown). Even though activity was slightly increased in all patient groups, no difference was seen between disease phases or between STEC-HUS and aHUS patients (data not shown). The lectin pathway was not investigated.

It is already known that STEC is able to manipulate complement factors in multiple ways: for instance, binding of Shiga toxin to FH results in a delayed cofactor activity on the cell surface, but this is not so in the fluid phase [16]; the autotransporter EspP was shown to cleave C3/C3b and C5 and decrease complement activation in serum [38]. We were surprised to see that in almost 30 % of STEC-HUS patients, a genetic complement aberration could be identified. Several case reports of STEC-HUS patients with complement mutations have been published [35, 39–42], but so far, only one larger cohort was investigated; in 3/25 (12.0 %) STEC-HUS patients, a mutation was identified [23].

In needs to be mentioned that the genetic aberrations identified in STEC-HUS patients are all variants of unknown significance. Most of the identified genetic variations have been described before as risk factors for other diseases associated with a dysregulated complement system (aHUS and/or age-related macular degeneration), and functional studies showed that these aberrations either influence the binding capacity of a complement to C3b regulators or affect the inactivation of C3 [5, 8–10, 43–45]. However, these were all reported in a (very) low frequency in the general population, and prediction software was not conclusive about their pathogenicity, which makes it more likely that these variants are disease modifying and not disease causing. This is not remarkable, as an incomplete penetrance is seen in HUS: healthy family members can carry disease-causing mutations [46]. This indicates that additional triggers, genetic and/or environmental, are probably needed for the disease to develop. It would be of great value to compare the level of complement aberrations in patients who develop HUS in a STEC outbreak to those that do not develop HUS, and see whether these numbers can be confirmed. A much larger study is warranted to determine the relevance of genetic variants in STEC-mediated HUS.

As in other studies, we could not link an altered complement profile in either the acute or convalescent phase of the disease to the presence of a genetic or acquired complement aberration (mutation or αFH; Electronic Supplementary Resource 1) [34]. Patients both with and without identified mutations displayed similar complement abnormalities regarding protein level, and not all patients with a complement aberration demonstrated complement activation for the analyzed biomarkers in the acute phase and/or in remission. These findings might also indicate that additional, as yet undiscovered, complement aberrations might play a role in some HUS patients with an abnormal serological complement profile. Moreover, abnormalities in complement regulation may only occur at the level of the endothelial cell surface and not systemically [34]. Therefore, serological levels of individual proteins may be normal in patients with complement dysregulation and thus cannot exclude a genetic complement disorder. The method used to monitor C3 and C5b–9 deposition on unstimulated human microvascular endothelial cells [34], however, is labor intensive and technically complex and can certainly not be performed in every clinic.

The finding of mutations and αFH in STEC patients argues that there might be undiagnosed cases of aHUS triggered by a STEC infection on a genetic background of impaired complement regulation, and that these might influence the development of disease after infection (only 5–15 % of infected individuals develop STEC-HUS). Unlike aHUS patients, so far, the investigated infection-induced HUS patients of our study with complement mutations or αFH had a good outcome and no recurrences. These patients, however, might be prone to develop recurrence. Indeed, one aHUS patient (P20) had a previous episode of HUS preceded by bloody diarrhea, but the recurrent episode had no evidence of a preceding STEC infection. In this patient, a *CD46* mutation was identified (Table 2). The same occurred in two Italian patients: they were first diagnosed with STEC-HUS, but after HUS recurrence, a complement mutation was identified [35]. Systematic follow-up of STEC-HUS patients, including genetic screening, is needed to learn more about the role of complement aberrations in STEC-HUS and to determine whether patients with STEC-HUS with mutations or αFH need to be grouped as aHUS patients instead.

Use of the complement inhibitor eculizumab in STEC-HUS is still questionable. Even though individual patients can respond rapidly with efficient recovery, in larger cohorts of STEC-HUS patients, no significant differences have been seen on mortality or morbidity with the use of eculizumab [47–50].

All together, we conclude that in both infection-induced HUS and aHUS patients the complement system is activated in the fluid phase during the acute phase of disease but not during remission. Measuring AP activation products, in particular C3d/C3 ratio, might help in monitoring disease activity and distinguishing between the different HUS etiologies. The exact role of altered complement activation in the pathogenesis of STEC-HUS, however, has not been fully elucidated. Interestingly, genetic and/or acquired complement aberrations (mutations and/or autoantibodies) were not only identified in aHUS patients but also in 30 % of infection-induced HUS patients. This indicates that genetic screening might be advised in these patients as well, as the presence of a genetic defect could influence the recurrence risk.

## Electronic supplementary material

Below is the link to the electronic supplementary material.

ESM 1 (PDF 307 kb)
