# Supplementary material for: Serological and genetic complement alterations in infection-induced and complement-mediated hemolytic uremic syndrome
Source: Pediatr Nephrol. 2016 Oct 7;32(2):297–309. doi: 10.1007/s00467-016-3496-0 (PMC5203860; doi:10.1007/s00467-016-3496-0)
Supplement: Supplementary file 1 — (PDF 307 kb) [file 467_2016_3496_MOESM1_ESM.pdf]

Article title: Serological and genetic complement alterations in infection-induced and complement mediated HUS

Journal: Pediatric Nephrology

Authors: Dineke Westra, Elena B. Volokhina, Renate G. van der Molen, Thea J.A.M. van der Velden, Annelies Jeronimus-Klaasen, Joop Goertz, Valentina Gracchi, Eiske M. Dorresteijn, Antonia H.M. Bouts, Mandy G. Keijzer-Veen, Johanna A.E. van Wijk, Jaap A. Bakker, Anja Roos, Lambert P. van den Heuve, Nicole C.A.J. van de Kar

Corresponding author: Dineke Westra, PhD., Dept. of Pediatric Nephrology, Radboud university medical center, Nijmegen, The Netherlands, dineke.westra@radboudumc.nl

Online Resource 1

Serological and genetic complement profile of HUS patients in the acute and convalescent phase of disease. Levels outside the control range for pediatric controls (defined by this study as mean ± 2 standard deviations) are indicated with (↑) or (↓). Patient 24 was on eculizumab in both convalescent phases; patient 8 and 24 had a second episode of aHUS.

Patient	Sex	Age	Follow up	Disease	Serotype	Acute phase									Convalescent phase									Genetics		αFH	αO157-LPS
						AP (%NHS)	C3 (mg/l)	FH (mg/l)	FI (%NHS)	C3d (mg/l)	C3d/C3 ratio	C3bBbP (AU/ml)	C3b/c (AU/ml)	TCC (AU/ml)	AP (%NHS)	C3 (mg/l)	FH (mg/l)	FI (%NHS)	C3d (mg/l)	C3d/C3 ratio	C3bBbP (AU/ml)	C3b/c (AU/ml)	TCC (AU/ml)	Mutations	CFH haplotype[a]		
P1	M	2y 7m	4y 5m	STEC-HUS	O26	90,1	594 (↓)	80 (↓)	36	20,78 (↑)	3,50 (↑)	6,67	17,62 (↑)	26,84 (↑)	106,7	1320 (↑)	280	75	15,86 (↑)	1,20 (↑)	11,66	38,31 (↑)	5,23	no aberrations	heterozygous	NEG	NEG
P4	F	10y 2m	4y 2m	STEC-HUS	O157	84,7	N/D	180	58	49,07 (↑)	N/D	N/D	N/D	27,14 (↑)	94,6	N/D	110	30	N/D	N/D	28,91 (↑)	49,69 (↑)	4,43	no aberrations	heterozygous	NEG	POS
P5	M	3y 8m	4y 1m	STEC-HUS	O26	112,7	937	240	93	N/D	N/D	N/D	N/D	N/D	120,0 (↑)	1440 (↑)	320	90	7,67	0,53	6,22	14,01	4,43	no aberrations	not present	NEG	NEG
P7	F	2y 3m	3y 11m	STEC-HUS	O157	117,5 (↑)	1630 (↑)	440	124	N/D	N/D	N/D	N/D	N/D	82,1	1140	270	75	6,89	0,60	14,03	20,94 (↑)	3,43	no aberrations	not present	NEG	NEG
P9	F	0y 10m	3y 7m	STEC-HUS	O104	91,5	N/D	150	50	N/D	N/D	N/D	N/D	N/D	89,5	1560 (↑)	280	79	8,01	0,51	15,22	28,86 (↑)	5,98	no aberrations	not present	POS	NEG
P10	M	11y 7m	3y 7m	STEC-HUS	O157	108,8 (↑)	1160	240	79	13,69 (↑)	1,18 (↑)	17,54	10,73	23,82 (↑)	103,3	1180	190	69	4,70	0,40	7,29	6,03	3,99	*no DNA available*		POS	POS
P12	M	8y 6m	3y 4m	STEC-HUS	O157	95,1	1060	220	73	11,15 (↑)	1,05	16,39	7,74	9,76 (↑)	69,9	930	180	75	4,13	0,44	106,88 (↑)	38,27 (↑)	5,68	no aberrations	not present	NEG	POS
P13	M	7y 7m	3y 4m	STEC-HUS	O157	90,3	1270	200	83	25,08 (↑)	1,98 (↑)	34,69 (↑)	65,09 (↑)	2,48	*No material available in convalescent phase of disease*									no aberrations	not present	NEG	POS
P14	F	2y 8m	3y 3m	STEC-HUS	O5	25,1	415 (↓)	170	51	10,98 (↑)	2,65(↑)	7,48	7,02	35,85 (↑)	103,4	N/D	360	99	3,68	N/D	10,25	10,11	4,77	*C3* : p.Arg1219His	not present	NEG	NEG
P15	M	3y 6m	3y 3m	STEC-HUS	O157	95,1	764	120	59	38,96 (↑)	5,10 (↑)	17,12	9,33	47,22 (↑)	114,5 (↑)	1270	290	101	5,59	0,44	15,72	10,32	4,97	no aberrations	heterozygous	NEG	NEG
P16	F	2y 0m	3y 3m	STEC-HUS	NT	104 (↑)	709	130	61	12,42 (↑)	1,75 (↑)	10,97	9,89	8,14 (↑)	102,1	928	190	59	12,42 (↑)	N/D	21,14 (↑)	16,15 (↑)	3,29	no aberrations	not present	NEG	NEG
P19	F	8y 4m	2y 8m	STEC-HUS	NT	97,2	1060	190	90	11,65 (↑)	1,10 (↑)	8,85	8,76	1,48	*No material available in convalescent phase of disease*									no aberrations	not present	NEG	NEG
P21	F	6y 3m	2y 7m	STEC-HUS	O157	*No material available in acute phase of disease*									N/D	1230	240	97	7,11	0,58	N/D	N/D	0,11	*CFH* : p.Thr956Met	not present	NEG	POS
P22	M	0y 6m	2y 7m	STEC-HUS	O157	93,7	626 (↓)	150	64	10,54 (↑)	1,68 (↑)	13,73	13,94	0,77	*No material available in convalescent phase of disease*									no aberrations	not present	NEG	POS
P23	M	1y 3m	2y 6m	STEC-HUS	O157	74,1	884	190	50	N/D	N/D	N/D	N/D	N/D	*No material available in convalescent phase of disease*									no aberrations	not present	NEG	POS
P25	F	2y 0m	2y 5m	STEC-HUS	O157	*No material available in acute phase of disease*									8,1	1310	340	81	6,44	0,49	10,87	9,27	4,79	*C3* : p.Lys155Gln	not present	NEG	POS
P26	M	11y 4m	2y 5m	STEC-HUS	NT	65,0	1140	200	63	13,61 (↑)	1,19 (↑)	24,00 (↑)	29,45 (↑)	15,38 (↑)	77,5	1040	220	39	4,14	0,40	4,89	5,38	6,47	no aberrations	not present	NEG	NEG
P27	F	0y 5m	2y	STEC-HUS	NT	88,0	953	370	92	6,78	N/D	13,52	11,21	2,40	66,3	710	140	63	N/D	N/D	N/D	N/D	N/D	no aberrations	not present	NEG	NEG
P28	M	0y 5m	2y	STEC-HUS	O26	59,5	462	180	39	N/D	N/D	N/D	N/D	N/D	67,6	813	250	48	7,59	N/D	11,21	18,67 (↑)	0,49	no aberrations	not present	NEG	NEG
P29	F	9y 4m	1y 11m	STEC-HUS	O26	90,2	801	230	78	9,09	N/D	14,36	13,39	6,02	42,6	913	230	65	4,64	0,51	6,88	9,96	2,97	*CFH* : p.Ser58Ala	heterozygous	NEG	NEG
P30	F	15y 3m	1y 11m	STEC-HUS	O157	42,7	750	140	48	17,10 (↑)	N/D	36,89 (↑)	15,36	3,53	70,8	1030	260	67	3,91	N/D	11,35	12,46	2,35	no aberrations	not present	NEG	POS
P32	M	17y 9m	2y	STEC-HUS	O157	81,2	1120	190	71	N/D	N/D	N/D	N/D	N/D	*No material available in convalescent phase of disease*									no aberrations	not present	POS	POS
P34	M	14y 4m	2y 3m	STEC-HUS	O157	99,4 (↑)	1160	260	70	3,87	N/D	21,03 (↑)	75,15 (↑)	8,72 (↑)	*No material available in convalescent phase of disease*									no aberrations	heterozygous	NEG	POS
P36	F	12y 11m	1y 5m	STEC-HUS	O157	95,2	810	N/D	N/D	21,72 (↑)	2,68 (↑)	7,34	17,91 (↑)	5,97	N/D	950	N/D	N/D	N/D	N/D	N/D	N/D	N/D	no aberrations	not present	NEG	POS
P37	F	12y 5m	1y 4m	STEC-HUS	O157	85,5	1340	N/D	N/D	6,07	N/D	7,29	11,70	0,44	N/D	1280	N/D	N/D	N/D	N/D	N/D	N/D	N/D	no aberrations	not present	NEG	POS
P38	M	3y 8m	1y 3m	STEC-HUS	O157	92,2	1010	N/D	N/D	20,15 (↑)	2,00 (↑)	12,83	18,77 (↑)	8,35 (↑)	67,0	864	N/D	N/D	4,18	N/D	7,41	10,49	3,29	no aberrations	not present	NEG	POS
P2	M	4y 2m	4y 4m	aHUS	-	95,6	N/D	230	65	N/D	N/D	N/D	N/D	4,38	101,1	2170	270	88	N/D	N/D	N/D	N/D	N/D	no aberrations	not present	NEG	NEG
P3	M	6y 8m	4y 3m	aHUS	-	69,5	N/D	180	60	N/D	N/D	N/D	N/D	N/D	N/D	1520	150	53	N/D	N/D	N/D	N/D	N/D	no aberrations	heterozygous	POS	NEG
P6	F	6y 4m	4y 1m	aHUS	-	91,1	970	270	81	N/D	N/D	N/D	N/D	N/D	*No material available in convalescent phase of disease*									no aberrations	heterozygous	NEG	NEG
P8	M	12y 7m	3y 11m	aHUS	-	96,3	110 (↓)	430	91	29,64 (↑)	26,95 (↑)	53,51 (↑)	125,24 (↑)	3,11	*No material available in convalescent phase of disease*									*C3* : p.Arg161Trp	not present	NEG	NEG
P11	F	7y 0m	3y 6m	aHUS	-	91,3	1130	330	115	11,56 (↑)	1,02	17,51	17,27 (↑)	2,00	*No material available in convalescent phase of disease*									no aberrations	not present	NEG	NEG
P18	F	11y 6m	2y 9m	aHUS	-	34,9	639	200	66	20,13 (↑)	3,15 (↑)	N/D	N/D	4,52	94,6	1190	240	58	6,40	0,54	7,39	10,02	5,58	no aberrations	not present	POS	NEG
P20	M	8y 2m	2y 8m	aHUS	-	74,7	657	150	41	N/D	N/D	26,60 (↑)	29,76 (↑)	2,35	*No material available in convalescent phase of disease*									*CD46* : p.Asp271_Ser272del	homozygous	NEG	NEG
P24	M	0y 6m	3y 4m	aHUS	-	74,1	1140	220	81	N/D	N/D	N/D	N/D	12,24 (↑)	N/D	1000	220	81	8,28	0,83	14,07	11,4	9,39 (↑)	no aberrations	homozygous	NEG	NEG
		1y 11m		aHUS	-	91,2	927	N/D	N/D	10,84 (↑)	1,17(↑)	19,93 (↑)	16,09 (↑)	1,21	N/D	899	N/D	N/D	7,39	0,74	14,75	10,48	4,52			NEG	NEG
P31	M	5y 7m	1y 11m	aHUS	-	2,7 (↓)	620 (↓)	160	56	20,39 (↑)	3,29 (↑)	35,12 (↑)	26,33 (↑)	13,43 (↑)	64,0	530 (↓)	180	46	N/D	N/D	N/D	N/D	N/D	no aberrations	not present	POS	NEG
P33	M	6y 4m	1y 11m	aHUS	-	62,1	645	200	52	N/D	N/D	N/D	N/D	N/D	*No material available in convalescent phase of disease*									*CD46* : p.Cys35Tyr	homozygous	NEG	NEG
P35	F	9y 11m	1y 11m	aHUS	-	92,9	850	270	96	27,53 (↑)	3,24(↑)	29,20 (↑)	43,70 (↑)	3,46	67,2	970	230	62	8,73	N/D	N/D	N/D	1,78	*C3* : p.Arg161Trp	not present	NEG	NEG
					Control range	24-99 %NHS	639-1312 mg/l	>97 mg/l	>19,5 %NHS	<9,73 mg/l	<1,08	<19,88 AU/ml	<15,92 AU/ml	<7,0 AU/ml	24-99 %NHS	639-1312 mg/l	>97 mg/l	>19,5 %NHS	<9,73 mg/l	<1,08	<19,88 AU/ml	<15,92 AU/ml	<7,0 AU/ml				

[a]Caprioli J, Noris M, Brioschi S, Pianetti G, Castelletti F, Bettinaglio P, Mele C, Bresin E, Cassis L, Gamba S, Porrati F, Bucchioni S, Monteferrante G, Fang CJ, Liszewski MK, Kavanagh D, Atkinson JP, Remuzzi G, International Registry of R, Familial HT (2006) Genetics of HUS: the impact of MCP, CFH, and IF mutations on clinical presentation, response to treatment, and outcome. Blood 108:1267-1279

αFH indicates 'autoantibodies against factor H'; aHUS 'atypical HUS'; AP 'alternative complement pathway'; AU 'arbitrary units'; C3 'complement component 3'; CD46 'membrane cofactor protein'; (C)FH 'complement factor H; (C)FI 'complement factor I'; F 'female'; HUS 'hemolytic uremic syndrome'; LPS 'lipopolysaccharide'; N/D 'not determined'; NEG 'negative'; NHS 'normal human serum'; M 'male'; POS 'positive'; SD 'standard deviation'; STEC-HUS 'Shiga-toxin producing Escherichia coli-induced HUS'; TCC 'terminal complement component'; (↑) 'increased compared to controls'; (↓) 'decreased compared to controls'.
